# The Effects of C4 Forage Silage with Different Water-Soluble Carbohydrate Contents on the Growth Performance, Apparent Digestibility, Rumen Fermentation, and Rumen Microbial Community of Buffaloes

**DOI:** 10.3390/ani16081233

**Published:** 2026-04-17

**Authors:** Qichao Gu, Jia Wang, Jie Zhang, Qiuxiang Ye, Zhiling Yan, Caixiang Wei, Xin Gao, Qi Yan, Yongqi Tan, Qingfeng Tang, Bo Lin, Xinghua Cai, Caixia Zou, Guangsheng Qin

**Affiliations:** 1 College of Animal Science and Technology, Guangxi University, Nanning 530004, China; guqchao@gmail.com (Q.G.); wangjia202303@126.com (J.W.); zhangj970271@163.com (J.Z.); ayeqiuxiang@163.com (Q.Y.); 15576333052@163.com (Z.Y.); 2218301034@st.gxu.edu.cn (C.W.); gaoxin961022@163.com (X.G.); yanqi7796@163.com (Q.Y.); renwoliu123@163.com (Y.T.); 899qinghui@163.com (Q.T.); linbo@gxu.edu.cn (B.L.); 13367811557@163.com (X.C.); 2 Guangxi Key Laboratory of Animal Breeding, Disease Control and Prevention, Nanning 530004, China; 3 Science and Technology Backyard of Guangxi Fusui Dairy Industry, Chongzuo 532100, China; 4 Buffalo Research Institute Chinese Academy of Agricultural Sciences and Guangxi Zhuang Nationality Autonomous Region, Nanning 530001, China

**Keywords:** *Bubalus bubalis*, water-soluble carbohydrates, silage quality, silage microbial communities, apparent digestibility, rumen fermentation, rumen microbial communities

## Abstract

Water-soluble carbohydrate (WSC) is the key to producing quality forage silage and an important energy source for ruminants. The aim of this study was to investigate the effect of different silages used as roughage sources [whole-plant sugarcane silage (WSS) vs. elephant grass silage (EGS)] with varying levels of WSC on silage quality, buffalo growth performance, apparent digestibility, rumen fermentation, and microbial communities. Sixteen healthy male crossbred buffaloes were randomly divided into two treatment groups, with eight buffaloes/treatment. One group was fed whole-plant sugarcane silage, and the other group was fed elephant grass silage. Compared with EGS, WSS had higher WSC, lactic acid, and ethanol, but lower pH, ammonia nitrogen, propionic acid, and butyric acid (BA) contents (*p* < 0.05). Potential probiotics (e.g., *Lactiplantibacillus* and *Hanseniaspora*) were more abundant in WSS than in EGS (*p* < 0.05). Moreover, the feed conversion rate was higher in HWS (*p* < 0.05). However, rumen fermentation parameters were unaffected by diet (*p* > 0.05). Moreover, feeding WSS had lower dry matter digestibility (DMD), organic matter digestibility (OMD), and lower acid detergent fiber digestibility (ADFD) (*p* < 0.05). After WSS feeding, ruminal *Treponema*_2 was strongly associated with DMD, OMD, and ADFD (*p* < 0.05), and also showed positive correlations with BA and PA contents in WSS (*p* < 0.05). Additionally, rumen *Ruminiclostridium*_5 and *Pseudozyma* was associated with DMD and ADFD after being fed EGS (*p* > 0.05), respectively, but the *Pseudozyma* was associated with BA (*p* < 0.05) and *Clostridium_sensu_stricto*_11 (*p* > 0.05) in EGS. Our findings indicated that WSS exhibited superior fermentation quality and harbored potential beneficial microbes, whereas EGS showed higher apparent nutrient digestibility in buffalo but also contained undesirable bacteria (e.g., *Clostridium_sensu_stricto*_11). Future research should investigate the long-term effects of WSS feeding on buffalo health, immunity, and production performance, as well as its impact on rumen microbiota stability, to fully assess its potential as a safe and sustainable roughage source.

## Simple Summary

Roughage quality affects ruminant health and production. Sugarcane and elephant grass are two common C4 plants in Guangxi, China, with different water-soluble carbohydrate (WSC) contents. This study compared sugarcane silage (WSS) and elephant grass silage (EGS) in buffaloes. WSS had better fermentation quality and a higher abundance of potential probiotics, but lower dry matter and organic matter digestibility. In contrast, EGS showed higher nutrient digestibility and lower feed conversion rate, though it contained potentially harmful bacteria. These findings provide a scientific basis for selecting C4 roughage with different WSC contents to improve feed utilization in buffaloes.

## 1. Introduction

Water buffaloes (*Bubalus bubalis*) efficiently utilize fibrous feeds [1]. China has the third largest number of water buffalo in the world, with approximately 23 million, and Guangxi, China, is the province with the largest number of water buffalo [2]. Guangxi’s climate is mainly subtropical monsoon, which is very suitable for the planting or growth of C4 forage [3]. Sugarcane (*Saccharum officinarum* L.) is widely cultivated as a cash crop in Guangxi, China, with a yield exceeding 70 million tons, primarily used for sugar production [4]. However, due to factors such as management methods and climate conditions, some sugarcane is of lower quality and unsuitable for sugar production [5]. This type of sugarcane can be considered for development as animal feed to achieve resource utilization [6]. Elephant grass (*Pennisetum purpureum*) is also widely distributed in Guangxi [4] and is a low-cost roughage. Silage is a common method for producing high-quality roughage in livestock, and its quality is significantly affected by the water-soluble carbohydrate (WSC) content of the raw materials. Sugarcane is rich in WSC, whereas elephant grass is low in WSC. Cavali et al. [7] compared the natural silage fermentation quality of sugarcane and elephant grass in a preliminary study and found that sugarcane silage had high WSCs, lactic acid (LA), or acetic acid (AA), low fiber content, and no butyric acid (BA), whereas elephant grass silage with low WSC content had propionic acid (PA), butyric acid (BA), low ethanol content and dry matter (DM) digestibility. However, it is not clear what the difference is in the silage quality between elephant grass and the low-quality sugarcane in Guangxi after ensiling. In addition, the core communities of bacteria and fungi need to be analyzed to identify the taxa causing differences in quality between the two silages, which will serve as a reference for the subsequent regulation of silage quality [8]. A recent study has shown that microorganisms (e.g., *Lactobacillus*) and metabolites (e.g., PA and BA) in silage have a positive impact on the metabolites and microorganisms present in the rumen [9].

Carbohydrates are the main source of energy in ruminants, and forage silages with differences in WSC content will have different effects when fed to ruminants. For example, forage silage with low-WSC content (e.g., elephant grass) can promote rumen microbial activity and enhance cellulose digestibility [10,11,12]. Compared with forage silage with low WSC content, forage silage with high WSC content may also increase palatability and nutrient digestibility, thereby improving intake and daily gain in ruminants. However, an increase in WSC content in forage silage leads to a decrease in rumen pH, which in turn reduces the activity of fiber-degrading bacteria and the rate of fiber degradation [13,14,15]. As a result, the residence time of chyme in the rumen increases, and animal feed intake is ultimately inhibited [16,17]. Moreover, forage silage with high WSC content can promote the synthesis of microbial protein and decrease ruminal NH3-N [18]. In addition, forage silage with high WSC content can increase the production of ruminal volatile fatty acids (VFAs), such as propionic acid (PA) [10,19]. Water buffalo, which play a vital role in agricultural production, have their production performance and health directly influenced by nutritional management [20]; however, the application of high-WSC roughage in their diet remains limited due to a lack of systematic data, restricting its broader use in practical farming.

Thus, the aim of the study was to compare the differences in C4 forage (elephant grass vs. sugarcane) silage quality based on low and high WSC contents and assess the effects of the differences in silage quality on growth performance, apparent nutrient digestibility, rumen fermentation, and the microbial community structure of buffaloes. We hypothesized that silage produced from the high-WSC forage sugarcane would have high WSC content, superior fermentation quality, and would be enriched in beneficial bacteria. Thus, when fed to buffaloes, this silage could improve growth performance, regulate the ruminal microbial community to reduce NH_3_-N, and enhance nutrient digestibility and rumen VFAs production.

## 2. Materials and Methods

### 2.1. Silage Preparation

In this experiment, the silage materials were harvested from the forage breeding farm of the Fusui Experimental Base of Guangxi University. The silage materials consisted of whole-plant sugarcane (Guitang No. 34) and elephant grass (Guimu No. 1) harvested at their mature growth stages. Fresh plant samples were crushed to 1–2 cm using a grinder (Model zfd5570, Zheng Feng Machinery Company, Jining, China) and then silaged using the wrapping method of Coblentz and Akins [21]. Chemical composition of the fresh elephant grass and whole-plant sugarcane is shown in Table 1. Ensiled at ambient temperature (24–30 °C) for 55 d, the silages were used to feed water buffaloes. A silage sample (20 g) was added to 180 mL of Ringer’s solution, mixed, and allowed to stand for 30 min at 4 °C. It was then filtered with double-layer gauze, and the filtrate was used to determine microbial communities and fermentation parameters.

### 2.2. Experimental Design

This study was conducted at the Guangxi Wokun Animal Husbandry Co., Ltd. (Nanning, China). Sixteen crossbred male water buffaloes (Age = 20 ± 2 months, weight = 527.4 ± 34.2 kg, mean ± SD) from the same farm were used in the experiment and were allocated into two groups. The control group was fed the basic diet supplemented with 50% elephant grass silage with low WSC content (LWE, *n* = 8), and the treatment group was fed the basic diet supplemented with 50% sugarcane silage with high WSC content (HWS, *n* = 8). Diet ingredients and nutrient levels are shown in Table 2.

The buffaloes were raised in sheds and fed each day at 0800 and 1600 during the experiment, with free access to food and water. The experiment lasted 46 d, consisting of 6 d of adaptation to the diet, followed by 40 d of experiment. Before and at the end of the experiment, all the buffaloes were weighed, and the live weights were used to calculate the daily body gain in weight. During the last 7 d of the experimental period, the dry matter intake (DMI) of each water buffalo was recorded daily. The feed conversion rate (FCR) was calculated as DMI divided by the average daily gain (ADG).

At the end of the experimental period, fresh fecal samples (200 g wet weight each) were collected from the rectum at 0800 and 1600 on 42 d, 43 d, 44 d, 45d, and 46, respectively. Samples from each animal were pooled for nutrient digestibility determination. Feed samples were collected daily during this period and stored at −20 °C for subsequent analysis. Apparent whole digestive tract nutrient digestibility was calculated using acid-insoluble ash as an internal standard, based on the concentration of acid-insoluble ash in the diet and feces.

Before the morning feeding on the last day of the experiment, seven water buffaloes were selected from each group for rumen fluid collection. Rumen fluid was collected by an oral stomach tube according to the method of Shen et al. [22]. The first 20 mL of rumen fluid was discarded, and then, 100 mL was extracted and divided into two parts: one part was frozen at –80 °C for determination of rumen microbial communities, and the other part was frozen at –20 °C for determination of NH_3_-N and VFAs, respectively. The pH was measured immediately.

### 2.3. Chemical Analysis

Samples (200 g) of forage silage, feed, and faces were analyzed for DM according to method 942.04 of AOAC [23]. After determination of DM, the samples were crushed with a grinder until they passed through a 40-mesh sieve and then used for determination of chemical composition and gross energy. Crude protein (CP) was determined using a Gerhardt fully automatic Kjeldahl nitrogen analyzer (method 988.05; K-375, Buchi, Uster, Switzerland) [23]. The method for determining crude ash is to ignite it in a muffle furnace at 550 °C for 4 h. The method for AIA is to treat the crude ash with hydrochloric acid and then ignite it again to constant weight. Water-soluble carbohydrates (WSCs) were determined using a sulfuric acid–anthrone colorimetric method [24]. Neutral detergent fiber (NDF) and acid detergent fiber (ADF) were determined by an Ankom-Fibre analyser 200i (ANKOM-Technology, Fairport, NY, USA) according to the method of Van Soest et al., with heat-stable alpha-amylase and sodium sulfite used in the NDF procedure [25]. Hemicellulose was calculated as the difference between NDF and ADF. Gross energy (GE) was determined using a Sundy SDC5015 oxygen bomb calorimeter (Hunan Sande Science and Technology Co., Ltd., Changsha, China). The following equation was used to calculate dry matter (DM) digestibility:

DM digestibility (%) = 100 − 100 × (AIA_diet_/AIA_feces_).

Apparent digestibility of other nutrients was then computed using the following equation:

Apparent nutrient digestibility (%) = 100 − (AIA_diet_/AIA_faces_) × (Nutrient_faces_/Nutrient_diet_) × 100

In which Nutrient_diet_ and Nutrient_faces_ represent the OM, CP, NDF, ADF, and GE concentrations in the diet and feces, respectively (on a DM basis).

According to the methods described by Gu et al. [8], the filtrates from silage were serially diluted for microbial counts (from 10^–1^ to 10^–6^). Lactic acid bacteria (LAB) were grown on De Man, Rogosa, and Sharpe agar (MRS, Beijing Land Bridge Technology, Beijing, China) and incubated at 35 °C for 48 h in an anaerobic incubator. Yeasts and molds were counted on potato dextrose agar (Beijing Land Bridge Technology) at 25 °C for 72 h. The number of enterobacteria (EB) was counted on violet-red bile agar (Beijing Land Bridge Technology) at 37 °C for 48 h.

### 2.4. Fermentation Index

The pH of the silage samples and rumen fluid was measured using a pH meter (Model DELTA 320, Mettler-Toledo Instrument Co. Ltd., Shanghai, China). The NH_3_-N in silage samples and rumen fluid was determined by a phenol–hypochlorite reaction method [26]. Volatile fatty acids (AA, PA, and BA) of silage samples and rumen fluid were determined using an Agilent-7890A gas chromatograph equipped with an HP-INNOwax capillary column (30.0 m × 0.320 µm × 0.50 µm, Agilent Technologies, Santa Clara, CA, USA) and a flame ionization detector. Briefly, at the time of sample injection, the injection volume was 1 µL, nitrogen was used as the carrier gas, and the temperature was increased from 60 °C to 200 °C at a rate of 20 °C/min, and then held at 200 °C for 3 min. The inlet and detector temperatures were 250 °C and 300 °C, respectively, and the split ratio was 10:1. The flow rate of hydrogen carrier gas was 25 mL/min.

### 2.5. DNA Extraction

The total DNA of each silage sample (20 g) and ruminal fluid sample (20 g) was extracted using an E.Z.N.A.^®^ Stool DNA Kit (Omega Biotek, Norcross, GA, USA) according to the manufacturer’s instructions. The integrity of extracted DNA was checked by 1% agarose gel electrophoresis, and DNA concentration and purity were determined with a NanoDrop 2000 UV-vis spectrophotometer (Thermo Scientific, Wilmington, DE, USA).

### 2.6. High-Throughput Sequencing

The extracted microbial DNA was sent to Shanghai Lingen Biotechnology Co., Ltd. (Shanghai, China) for 16S rRNA and ITS1 gene amplification. For the bacterial community, the V3-V4 region of the 16S rRNA gene was amplified using the universal bacterial primers 341F (CCTAYGGGRBGCASCAG) and 806R (GGACTACHVGGGTWTCTAAT). For the fungal community, the primers ITS5-1737F (GGAAGTAAAAGTCGTAACAAGG) and ITS2-2043R (GCTGCGTTCTTCATCGATGC) were used to amplify the fungal ITS1 region. The PCR products were mixed in equimolar ratio and purified by using a QIAquick Gel Extraction Kit (Qiagen, Dusseldorf, Germany). All PCR mixtures contained 15 μL of Phusion High-Fidelity PCR Master Mix (Thermo Fisher, Waltham, MA, USA), 0.2 μM primers, and 10 ng of genomic DNA template. The first denaturation was performed at 98 °C for 1 min, followed by 30 cycles at 98 °C (10 s), 50 °C (30 s), and 72 °C (30 s), and then finally maintained at 72 °C for 5 min. The sequence library was constructed with a TruSeq^®^ DNA PCR-Free Sample Preparation Kit (Illumina Inc., San Diego, CA, USA), and sequencing was performed using the Illumina NovaSeq 6000 platform (Illumina Inc., San Diego, CA, USA).

### 2.7. Analysis of Microbial Diversity

Uparse software (v7.0.1001, http://drive5.com/uparse/, accessed on 5 May 2024) was used to cluster sequences of valid sample data at 97% similarity to form operational taxonomic units (OTUs) [27]. The Silva (http://www.arb-silva.de/, accessed on 10 May 2024) and UNITE databases (https://unite.ut.ee/, accessed on 12 May 2024) were used to annotate the bacterial and fungal sequencing data, respectively, to obtain taxonomic information and calculate the community composition of each sample at each taxonomic level (i.e., kingdom, phylum, class, order, family, genus, and species). The alpha diversity indices, observed species, Shannon’s, and Simpson’s, were calculated using Qiime 2 software (v2024.10) [28]. Linear discriminant analysis effect size (LEfSe) software (v1.0) was employed to compare differences in bacterial and fungal communities between groups [8]. The key microbial taxa were selected for Spearman correlation analysis with rumen fermentation parameters based on the LEfSe analysis. The microbial community structure, alpha diversity, and the correlation matrix between key microbial taxa and rumen fermentation parameters were drawn using the “ggpolt2” package in RStudio (v4.0.3) [28].

### 2.8. Statistical Analyses

Data were analyzed with SPSS 19.0 (SPSS Inc., Chicago, IL, USA), and Student’s *t*-test was used to identify significant differences between treatment means, with *p* < 0.05 considered significant and 0.05 ≤ *p* < 0.10 indicating a tendency.

## 3. Results

### 3.1. Chemical Composition and Fermentation Quality of Silage

The CP and WSC contents in WSS were significantly higher than those in EGS (*p* < 0.05; Table 3), whereas the DM content was significantly lower in WSS (*p* < 0.05). The NDF, ADF, and hemicellulose contents were not significantly different between WSS and EGS (*p* > 0.05). In addition, EB counts in WSS were significantly higher than those in EGS (*p* < 0.05), but yeast counts in WSS were significantly lower than those in EGS (*p* < 0.05), although those counts were less than 4.0 log colony-forming units (CFU)/g fresh matter (FM). The pH, PA, and BA contents were significantly higher in EGS (*p* < 0.05), while LA and ethanol contents were significantly lower in EGS (*p* < 0.05).

### 3.2. Alpha Diversity of Silage Microbial Communities

**Figure 1:**
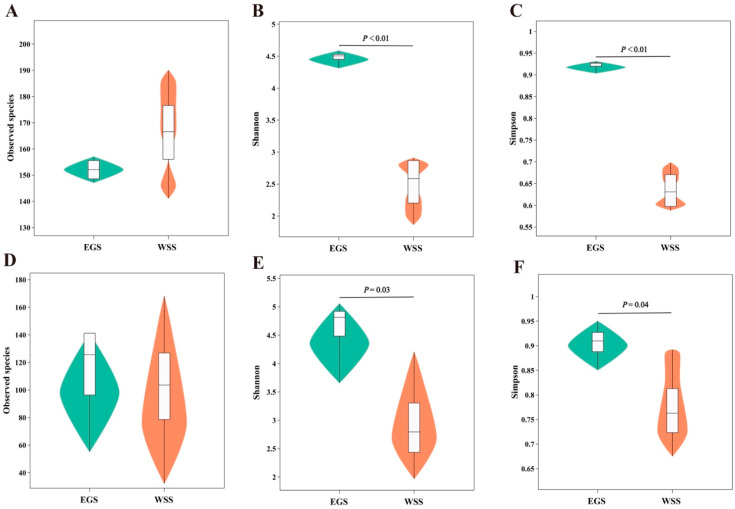
Alpha diversity indices of (**A**–**C**) bacterial and (**D**–**F**) fungal communities in elephant grass silage (EGS) and whole-plant sugarcane silage (WSS). The central box indicates the interquartile range; the thin black line represents the 95% confidence interval; the black horizontal line within each box is the median; and the outer shape indicates the density of the data distribution. *p* < 0.05 is considered statistically significant.

Bacterial and fungal alpha diversity indices are shown Figure 1. The observed species of the silage bacteria (Figure 1A) and fungi (Figure 1D) were not significantly different between EGS and WSS. However, the Shannon and Simpson indices of silage bacteria (Figure 1B,C) and fungi (Figure 1E,F) were significantly higher in EGS than in WSS (*p* < 0.05). Moreover, the library coverage of bacteria and fungi in all samples from the two silages was greater than 99% (Table S1).

**Figure 2:**
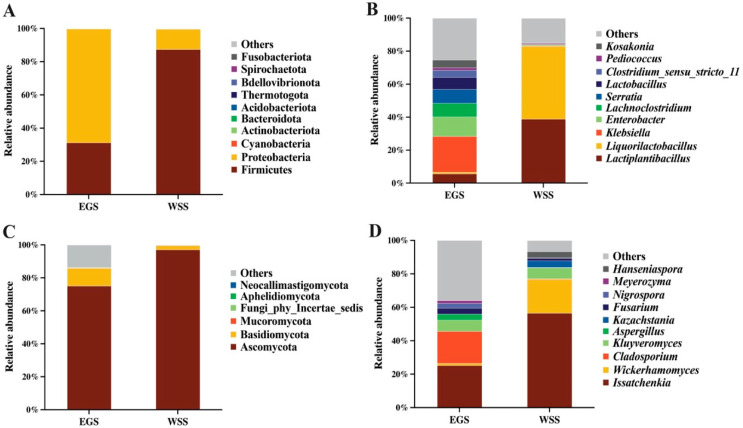
Relative abundances of bacterial (**A**,**B**) and fungal (**C**,**D**) phyla and genera of elephant grass silage (EGS) and whole-plant sugarcane silage (WSS).

The relative abundances of phyla and genera of silage bacteria are shown in Figure 2A,B. The two most abundant phyla in both groups were *Firmicutes* (EGS = 31.22%, WSS = 87.37%) and *Proteobacteria* (EGS = 68.56%, WSS = 12.22%) (Figure 2A). The two most abundant genera in EGS were *Klebsiella* (21.68%) and *Enterobacter* (11.88%), whereas in WSS, *Lactiplantibacillus* (38.78%) and *Liquorilactobacillus* (44.24%) were the two most abundant genera (Figure 2B). The relative abundances of phyla and genera of silage fungi are shown in Figure 2C,D. The two most abundant phyla in both groups were *Ascomycota* (EGS = 75.12%, WSS = 97.04%) and *Basidiomycota* (EGS = 10.79%, WSS = 2.55%) (Figure 2C). The two most abundant genera in EGS were *Issatchenkia* (29.21%) and *Cladosporium* (19.15%), whereas in WSS, *Issatchenkia* (56.54%) and *Wickerhamomyces* (20.30%) were the two most abundant genera (Figure 2D).

### 3.3. Analysis of Variation in Silage Bacteria and Fungi

**Figure 3:**
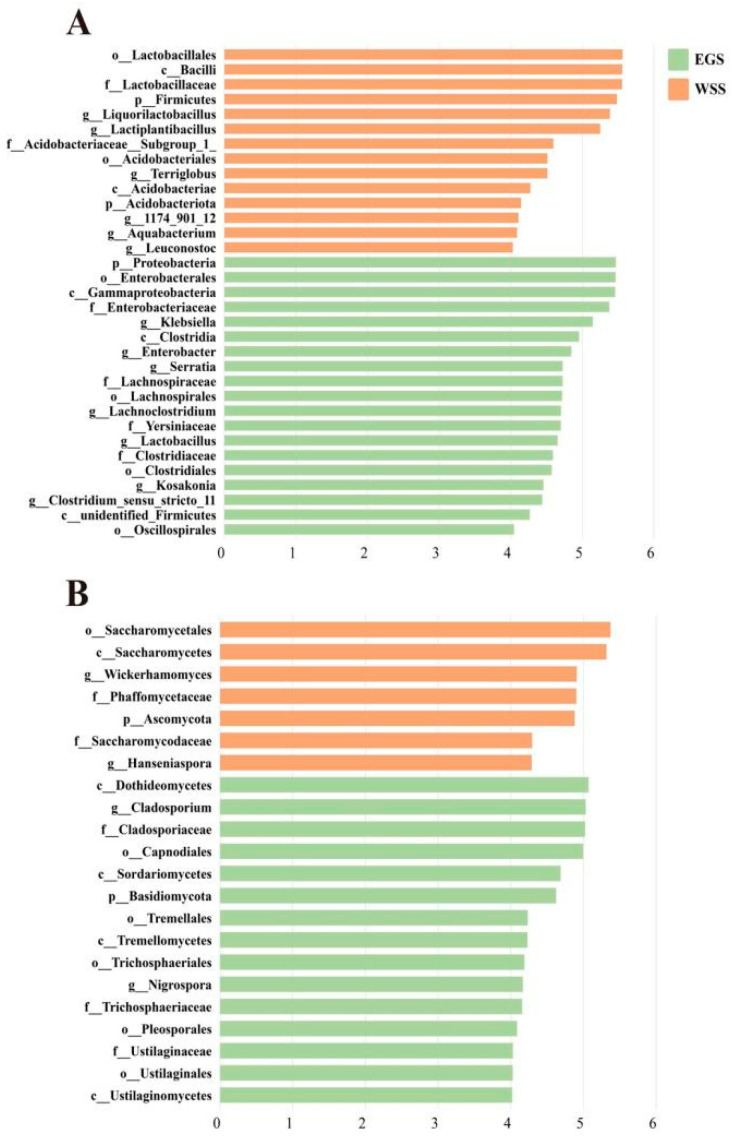
Linear discriminant analysis (LDA) effect size (LEfSe) analysis of significantly different taxa of (**A**) bacteria and (**B**) fungi in elephant grass silage (EGS) and whole-plant sugarcane silage (WSS). Taxa are ranked by LDA score.

Differences in the bacterial and fungal communities between silages were analyzed using the LEfSe method (Figure 3A,B). The bacterial and fungal taxa with an LDA score > 4 were defined as unique. For bacteria, *Lactobacillus*, *Kosakonia*, *Serratia*, *Klebsiella*, *Enterobacter*, *Clostridium_sensu_stricto_11*, and *Lachnoclostridium* were more important in EGS than in WSS (Figure 3A), whereas *Leuconostoc*, *Aquabacterium*, *Lactiplantibacillus*, *Liquorilactobacillus*, and *Terriglobus*, and *1174_901_12* were more important in WSS than in EGS (Figure 3A). For fungi, *Nigrospora* and *Cladosporium* were more important in EGS than in WSS, whereas *Hanseniaspora* and *Wickerhamomyces* were more important in WSS than in EGS (Figure 3B).

### 3.4. Feed Intake, Animal Performance, Apparent Nutrient Digestibility, and Rumen Fermentation

The ADG in LWE was higher than that in HWS, with no significant difference (*p* > 0.05; Table 4). The DMI in LWE was significantly higher than that in HWS (*p* < 0.05). While, the FCR were significantly higher in HWS than that in LWE (*p* < 0.05). The DM digestibility (DMD), OM digestibility (OMD), and ADF digestibility (ADFD) were significantly lower in HWS (*p* < 0.05). The digestibility of CP, NDF, and GE were not significantly different between HWS and LWE (*p* > 0.05). Ruminal pH and NH_3_-N, AA, PA, and BA contents were also not significantly different (*p* > 0.05) between LWE and HWS.

### 3.5. Rumen Microbial Diversity and Community

**Figure 4:**
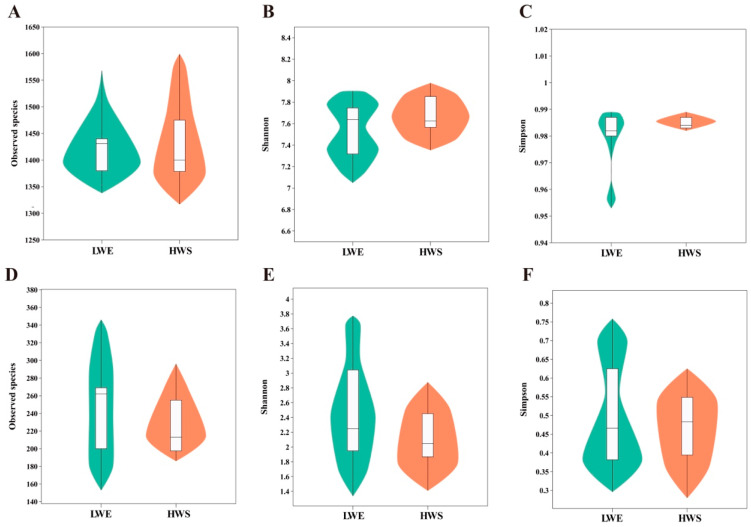
Alpha diversity indices of (**A**–**C**) bacterial and (**D**–**F**) fungal communities in the ruminal fluid of water buffaloes fed diets containing elephant grass silage with low WSC content (LWE) or whole-plant sugarcane silage with high WSC content (HWS). The central box indicates the interquartile range; the thin black line represents the 95% confidence interval; the black horizontal line within each box is the median; and the outer shape indicates the density of the data distribution.

The alpha diversity indices of bacteria (Figure 4A–C) and fungi (Figure 4D–F) of number of observed species, Shannon’s, and Simpson’s in rumen fluid were not significantly different (*p* > 0.05) between LWE and HWS. The library coverage of bacteria and fungi in all samples from the two groups was greater than 99% (Table S2).

**Figure 5:**
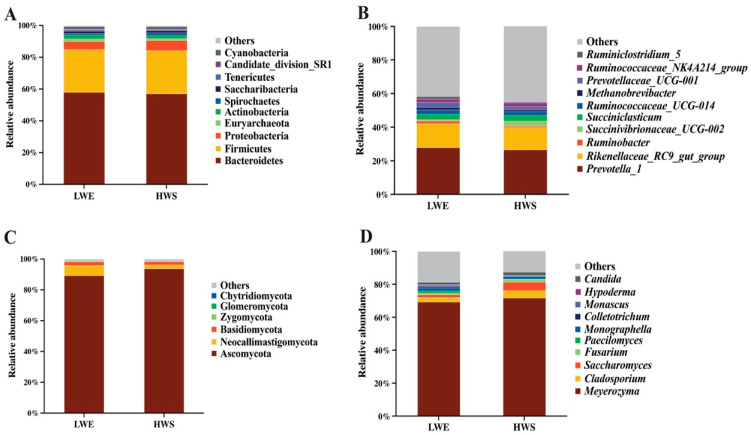
Relative abundances of bacterial (**A**,**B**) and fungal (**C**,**D**) phyla and genera in the ruminal fluid of water buffaloes fed diets containing elephant grass silage with low WSC content (LWE) or whole-plant sugarcane silage with high WSC content (HWS).

The relative abundances of phyla and genera of bacteria in rumen fluid are shown in Figure 5A,B. In general, feeding forage silage with different WSC content had limited effects on the 10 most abundant phyla and genera of the bacterial community in rumen fluid. The three most abundant phyla were similar in both groups, which were *Bacteroidetes* (LWE = 57.97%, HWS = 57.07%), *Firmicutes* (LWE = 26.87%, HWS = 27.19%), and *Proteobacteria* (LWE = 5.14%, HWS = 6.39%) (Figure 5A). The three most abundant genera were *Prevotella* 1 (LWE = 26.89%, HWS = 26.63%), *Rikenellaceae RC9 gut group* (LWE = 14.29%, HWS = 13.26%), and *Succiniclasticum* (LWE = 3.18%, HWS = 3.38%) (Figure 5B).

The relative abundances of phyla and genera of fungi in rumen fluid are shown in Figure 5C,D. Feeding forage silage with different WSC content also had limited effects on the 10 most abundant phyla and genera of the fungal community in rumen fluid. The most common phyla in both groups were *Ascomycota* (LWE = 89.30%, HWS = 93.76%), *Neocallimastigomycota* (LWE = 6.61%, HWS = 2.51%), and *Basidiomycota* (LWE = 2.36%, HWS = 2.12%) (Figure 5C). The five most abundant genera were *Meyerozyma* (LWE = 69.27%, HWS = 71.71%), *Cladosporium* (LWE = 2.70%, HWS = 4.51%), *Saccharomyces* (LWE = 1.75%, HWS = 5.09%), *Monographella* (LWE = 1.84%, HWS = 1.70%), and *Candida* (LWE = 1.43%, HWS = 2.16%) (Figure 5D).

### 3.6. Analysis of Variation in Rumen Bacteria and Fungi

**Figure 6:**
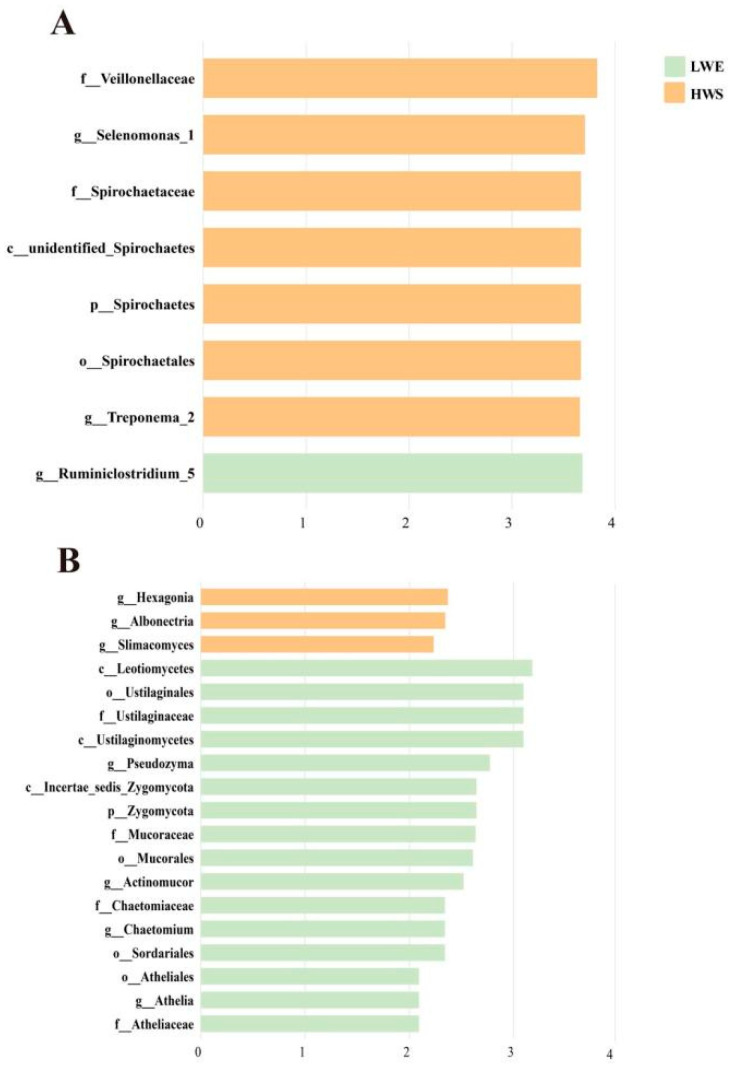
Linear discriminant analysis (LDA) effect size (LEfSe) analysis of significantly different taxa of ruminal (**A**) bacteria and (**B**) fungi in the ruminal fluid of water buffaloes fed diets containing elephant grass silage with low WSC content (LWE) or whole-plant sugarcane silage with high WSC content (HWS). Taxa are ranked by LDA score.

To better understand the dominance of specific bacteria within the two silages, we used LEfSe analysis (Figure 6A). Bacteria with an LDA score > 3 were defined as unique. The genus *Ruminiclostridium_*5 (*p* = 0.03) was significantly enriched in LWE, whereas the genera *Treponema_*2 (*p* < 0.01) and *Selenomonas_*1 (*p* < 0.01) were significantly enriched in HWS, and these bacterial genera had a significant effect on sample grouping.

The dominance of specific fungi within the two silages was also explored by using LEfSe analysis (Figure 6B). Fungi with an LDA score > 2 were defined as unique. The genera *Athelia* (*p* < 0.01), *Chaetomium* (*p* = 0.05), *Actinomucor* (*p* < 0.01), and *Pseudozyma* (*p* = 0.03) were significantly enriched in LWE, whereas *Slimacomyces* (*p* = 0.01), *Albonectria* (*p* = 0.02), and *Hexagonia* (*p* = 0.03) were significantly enriched in HWS, and these fungal genera had a significant effect on sample grouping.

### 3.7. Mantel Correlation Analysis

**Figure 7:**
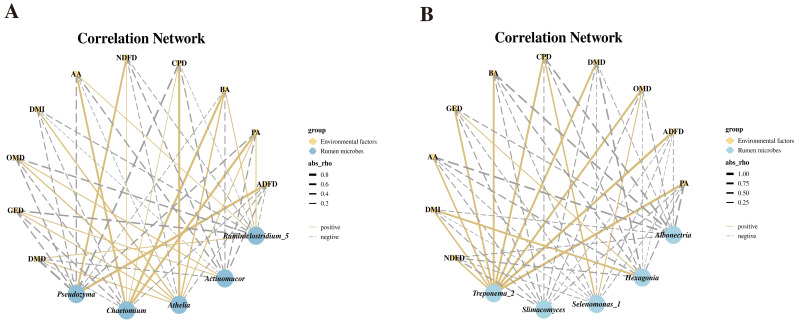
Correlations between key bacterial and fungal taxa in the rumen and ruminal fermentation parameters and apparent nutrient digestibility in LWE (**A**) and HWS (**B**). LWE, water buffaloes fed diets containing elephant grass silage; HWS, water buffaloes fed diets containing whole-plant sugarcane silage; AA, acetic acid; PA, propionic acid; BA, butyric acid; DMI, dry matter intake; DMD, dry matter digestibility; CPD, crude protein digestibility; NDFD, neutral detergent fiber digestibility; ADFD, acid detergent fiber digestibility, OMD, organic matter digestibility; GED, gross energy digestibility.

Mantel analysis shows the correlation between biomarker microbial taxa and DMI, rumen fermentation parameters, and apparent digestibility of various nutrients is presented in Figure 7A,B. In LWE, only *Pseudozyma* was strongly positive correlated with NDFD (*p* > 0.05), and in ADFD (*p* > 0.05), *Ruminiclostridium_*5 was strongly positive correlated with DMD (*p* > 0.05) (Figure 7A). In HWS, *Treponema_2* was strongly positive correlated with ruminal AA (*p* < 0.05), PA (*p* < 0.01), BA contents (*p* < 0.05), DMD (*p* < 0.01), CPD (*p* < 0.01), ADFD (*p* < 0.01), and GED (*p* < 0.01). *Treponema_2* and *Selenomonas_*1 were strongly positive correlated with OMD (*p* < 0.01) (Figure 7B).

### 3.8. Correlation Analysis Between Key Rumen Microbial and Metabolites and Key Microbial in Silage

**Figure 8:**
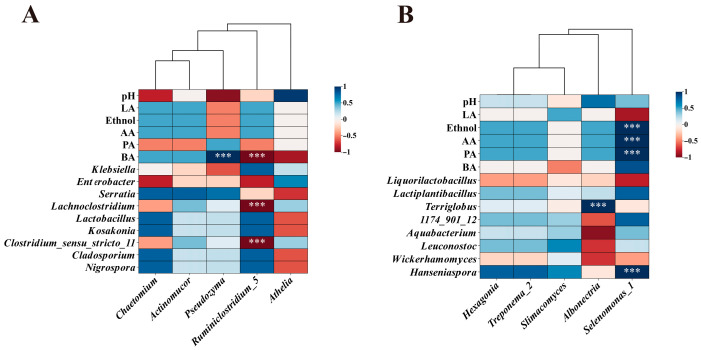
Correlation between key rumen bacterial and fungal taxa in LWE (**A**) and HWS (**B**) and biomarker microorganisms and metabolites in EGS and WSS, respectively. LWE, water buffaloes fed diets containing elephant grass silage; HWS, water buffaloes fed diets whole-plant sugarcane silage; AA, acetic acid; PA, propionic acid; BA, butyric acid. Blue indicates a perfect negative correlation, while red indicates a perfect positive correlation. *** *p* < 0.001.

A Spearman correlation matrix between mean abundances of key bacterial and fungal taxa in the rumen and key bacterial and fungal taxa and metabolites in the silage is presented in Figure 8A,B. In LWE, Ruminal *Pseudozyma* was negative correlated with pH (*p* > 0.05), LA (*p* > 0.05), ethanol (*p* > 0.05), AA contents (*p* > 0.05), *Klebsiella* (*p* > 0.05) and *Enterobacter* (*p* > 0.05) in silage, but it was positive correlated with PA (*p* > 0.05), BA contents (*p* < 0.01), *Serratia* (*p* > 0.05)*, Lachnoclostridium* (*p* > 0.05), *Lactobacillus* (*p* > 0.05)*, Kosakonia* (*p* > 0.05), *Clostridium_sensu_stricto_11* (*p* > 0.05), *Cladosporium* (*p* > 0.05), and *Nigrospora* (*p* > 0.05) in silage (Figure 8A). In HWS, Ruminal *Treponema_2* was negative correlated with *Liquorilactobacillus* (*p* > 0.05) and *Wickerhamomyces* (*p* > 0.05) in silage, but it was strongly positive correlated with ethnol (*p* > 0.05), AA (*p* > 0.05), PA contents (*p* > 0.05), *Lactiplantibacillus* (*p* > 0.05), *Terriglobus* (*p* > 0.05), *1174_901_12* (*p* > 0.05), *Aquabacterium* (*p* > 0.05), *Leuconostoc* (*p* > 0.05), and *Hanseniaspora* (*p* > 0.05) in silage (Figure 8B). *Selenomonas_*1 was negative correlated with LA content (*p* > 0.05), *Liquorilactobacillus* (*p* > 0.05) and *Wickerhamomyces* (*p* > 0.05) in silage, but it was strongly positive correlated with ethnol (*p* < 0.01), AA (*p* < 0.01), PA (*p* < 0.01), BA contents (*p* > 0.05), *Lactiplantibacillus* (*p* > 0.05)*, 1174_901_12* (*p* > 0.05), *Aquabacterium* (*p* > 0.05), *Leuconostoc* (*p* > 0.05), and *Hanseniaspora* (*p* < 0.01) in silage (Figure 8B).

## 4. Discussion

### 4.1. Fermentation Quality of Silage

Assessing the chemical composition of silage is the most fundamental and crucial step in the entire silage quality evaluation system [29]. In this study, WSS had significantly higher WSC and CP contents than those of EGS. These results were mainly due to the higher WSC and CP contents in fresh whole-plant sugarcane than in elephant grass (Table S1). However, Cavali et al. [7] reported that compared with sugarcane silage, CP and WSC contents were higher in elephant grass silages, whether with or without bacterial inoculant, which might be due to differences in forage varieties, harvesting time, growing environment, and management, and so on. Similar results are reported by Cavali et al. [7], EGS also had higher DM content in this study. This result is also attributed to the higher DM content in fresh elephant grass (Table S1). Structural carbohydrates affect animal digestion, utilization, and production performance [28]. Compared with EGS, WSS had higher EB and lower yeast counts in this study. The reason why EB were enriched in WSS might be because of some acid-resistant species of EB [30]. The reason for the decrease in number of yeasts might be because the pH of WSS was 3.39, which is not the optimal pH range (i.e., 4–6) for yeast growth [31], thus limiting its proliferation. Notably, both EB and yeasts are considered undesirable microorganisms in silage. However, the counts of EB and yeasts were all less than 4 log_10_ CFU/g FM in the two silages. Studies have pointed that keeping the counts of yeast and EB at 4 log_10_ CFU/g FM or below helps ensure the fermentation quality of silage [32,33].

Silage pH is one of the important indicators of silage quality. In the present study, the pH in WSS was lower than that in EGS. This result is mainly because the whole sugarcane plant provides more sugar substrates for lactic acid bacteria to produce LA and thus lowering the pH [34]. That is why LA content was higher in WSS than in EGS (Table 2). LA can effectively inhibit the growth of harmful microorganisms in silage under anaerobic conditions [35]. Consistent with previous studies on sugarcane silage [36,37], this study also found higher ethanol content in WSS. This result is mainly because yeasts can use the residual WSC in sugarcane to produce ethanol. Compared with LA, PA can effectively inhibit the growth of harmful bacteria under aerobic fermentation [38]. In this study, the PA content, as well as the BA content, was higher in EGS than in WSS. However, an increase in BA content in silage may stimulate the growth of unfavorable microorganisms and decrease silage quality [39]. The NH_3_-N content mainly reflects the degree of protein decomposition in silage by microorganisms [40]. In present study, the NH_3_-N content was higher in EGS than in WSS, likely due to the presence of protein-decomposing bacteria, such as EB, in silage [41].

### 4.2. Microbial Communities of Silage

The Shannon and Simpson indices represent the richness and evenness, respectively, microbial communities. In this study, Shannon and Simpson indices of bacteria were lower in WSS than in EGS. These results are possibly due to the lower pH in WSS than in EGS, which limited the growth of acid-intolerant bacteria. In the present study, the dominant phyla of bacteria were *Firmicutes* and *Proteobacteria* in both WSS and EGS. Similar results are also found in elephant grass silage and other high-WSC content forage silages [8,28]. *Firmicutes* and *Proteobacteria* can degrade complex organic compounds under anaerobic conditions, including proteins and cellulose [42,43]. According to Keshri et al. [32,44], low pH under anaerobic conditions favors the reproduction of *Firmicutes* species. Thus, in this study, the relative abundance of *Firmicutes* was higher in WSS than in EGS, with abundance exceeding 80%. *Klebsiella* and *Enterobacter* were dominant genera in EGS. Similarly, *Klebsiella* and *Enterobacter* are the dominant bacterial genera in natural elephant grass silage [45,46] and wilted elephant grass [47], respectively. However, similar results are not found in other natural elephant grasses, perhaps because of differences in varieties [48]. Both *Klebsiella* and *Enterobacter* are in the family *Enterobacteriaceae*, which breaks down lactic acid to produce other substances and includes harmful bacteria in silage [49]. In WSS, the dominant genera were *Lactiplantibacillus* and *Liquorilactobacillus*. *Lactiplantibacillus* is also the dominant bacterial genera in *Caragana korshinskii* silage and *Triticale* silage [50,51]. However, there are few reports on *Liquorilactobacillus* in silage. Notably, species of lactic acid bacteria in *Lactiplantibacillus* and *Liquorilactobacillus* can decompose and utilize the sugar in sugarcane to produce lactic acid under anaerobic conditions [52]. Furthermore, in this study, LEfSe analysis showed that genera from *Clostridiaceae* (*Clostridium_sensu_stricto_11*) and *Enterobacteriaceae* (*Kosakonia*, *Serratia*, *Klebsiella*, *Enterobacter*) were more likely to proliferate in EGS, while in WSS, genera from *Lactobacillaceae* (*Leuconostoc*, *Lactiplantibacillus*, and *Liquorilactobacillus*) were more likely to proliferate. Generally, *Clostridiaceae* and *Enterobacteriaceae* are the main families that lead to poor silage quality. The possible reason is that the environment is suitable for the proliferation of those genera of bacteria in EGS (silage pH > 5.0). By contrast, *Lactobacillaceae*, containing lactic acid bacteria, is one of the key bacterial groups in producing high-quality fermented feed. The reason is that sugarcane can increase the WSC content for lactic acid bacteria to produce LA, creating an acidic environment and promoting their growth.

In this study, the Shannon and Simpson indices of fungi were lower in WSS than in EGS. These results might also be because of lower pH in WSS than in EGS, which limited the growth of acid-intolerant fungi. However, *Ascomycota* and *Basidiomycota* were the dominant fungal phyla in both WSS and EGS. Similar results are also found in elephant grass silage and other high-WSC content forage silages [8,28,48,53]. At the genus level, *Issatchenkia* was the dominant genus in both silages in this study, and consistent with this result, Guan et al. [54] and Liu et al. [55] found that *Issatchenkia* was the most abundant genus in Napier grass silage and barley silage, respectively. However, Liu et al. [55] report that *Issatchenkia* is the main yeast that causes aerobic spoilage. The second most dominant genus in EGS was *Cladosporium*. *Cladosporium* can produce mycotoxins, leading to reduced silage quality [56]. Consistent with the results of this study, *Wickerhamomyces* was the second most dominant genus in high-WSC sugarcane silage [8]. Some species of *Wickerhamomyces* secrete mycotoxins that have bactericidal effects [57]. In addition, LEfSe analysis showed that species of mycotoxin-producing fungi (*Nigrospora* and *Cladosporium*) were more likely to reproduce in EGS [55,58]. This result might be because the silage pH (>5.0) in EGS is suitable for growth of these fungi. However, in WSS, genera of fungi with antimicrobial properties (*Hanseniaspora* and *Wickerhamomyces*) were more likely to reproduce [57,59], which might be because sugarcane provided a rich source of WSCs for their growth. Astuti et al. [60] pointed out that *Hanseniaspora* has the potential for probiotic applications, including improving the nutritional value and sensory properties of fermentation substrates.

### 4.3. Rumen Fermentation and Growth Performance

In this study, the rumen fermentation parameters of pH and NH_3_-N, AA, PA, and BA contents were not different between LWE and HWS. Moreover, in the present study, whether the diet was WSS- or EGS-based had no significant effects on DMI. Suong et al. [61] also found that fermentation parameters and DMI were not affected after feeding on black cane silage and Napier grass silage. These results may be due to the fact that buffalo can efficiently process a wide range of forage silages. The FCR was higher in HWS. However, previous studies have shown that increasing dietary non-fiber carbohydrates can improve FCR in growing buffaloes [62]. In contrast, the present study found that feeding high-WSC silage to adult buffaloes led to an increase in FCR. This discrepancy may be attributed to differences in sugar composition and animal physiological stage. Notably, adult late-stage buffaloes (520–550 kg) exhibit a high FCR, largely because energy intake is prioritized for maintenance, constraining weight gain potential [63,64]. Accordingly, FCR were high in both the LWE and HWS groups. In addition, the DMD and OMD were lower in HWS. This may be that the low pH of WSS inhibits the microorganisms involved in nutrient digestibility in the rumen (e.g., fiber-degrading bacteria) [65]. However, previous studies have reported that lactating dairy cows or Holstein heifers fed diets based on high-WSC forage (e.g., early-harvested perennial ryegrass silage, and sweet sorghum silage) silage had higher DMD and OMD [14,66]. This discrepancy may be due to differences in forage varieties and animal species. Moreover, the low silage pH of WSS may also have inhibited specific fiber-degrading bacteria in the rumen (e.g., *Ruminococcus albus* and *Fibrobacter succinogenes*), which explains why ADFD was lower in HWS. While, this may be due to differences in forage varieties, Benchaar et al. [67] found that ruminants had higher ADFD after being fed diets based on high-WSC forage silage (e.g., red clover silage and sweet sorghum silage). Notably, although CPD, NDFD, and GED were higher in the LWE than in the HWS, the difference was not significant. This may still be related to the inhibitory effect of the enriched metabolites (e.g., ethanol) and competitive microorganisms in WSS on relevant functional rumen microorganisms.

### 4.4. Microbial Diversity and Community Structure of the Buffalo Rumen

In the present study, the dominant bacterial phyla in the rumen of buffaloes fed forage silage with either low or high WSCs content were *Bacteroidetes*, *Firmicutes*, and *Proteobacteria*. Similar results are also found in the rumen bacterial communities of cattle and buffaloes [68,69]. In addition, in this experiment, the dominant bacterial genera in the rumen of buffaloes fed forage silage of either low or high WSCs content were *Prevotella 1*, the *Rikenellaceae* RC9 gut group, and *Succiniclasticum*. Similarly, *Prevotella* is the dominant genus in the rumen of buffalo in another study [70]. Ruminal *Prevotella* spp. generally ferment hemicellulose, starch, protein, peptides, and pectin into succinate, propionate, and acetate [71,72]. The *Rikenellaceae* RC9 gut group is the most abundant group also found in cattle [73]. This genus is involved in fiber degradation [74]. The genus *Succiniclasticum* is a constituent among the core rumen microflora, capable of converting succinate into propionate [75,76]. *Ruminiclostridium*_5 was enriched in LWE. *Ruminiclostridium* species are recognized for their capacity to degrade complex plant fibers, including cellulose and hemicellulose, via cellulosomal multi-enzyme complexes [77]. Moreover, the Mantel test showed positive correlations between *Ruminiclostridium*_5 and DMD. This may explain why higher nutrient digestibility (e.g., DMD) was observed in the LWE group. Moreover, the relative abundances of *Treponema_2* were higher in HWS than in LWE. *Treponema_2* are cellulolytic bacteria [78] and it is positively correlated with AA, PA, and BA contents in the rumen [79]. The Mantel test showed positive correlations between *Treponema_2* and the AA, PA, and BA contents. However, this genus may represent low-abundance bacteria, as the AA, PA, and BA contents in the HWS did not increase significantly (Table 3). The Mantel test showed a positive correlation between *Treponema_2* and DMD and OMD. Moreover, McLoughlin et al. (2020) have pointed that *Treponema_2* exhibited significant negative associations with ADG [80]. This may be why the ADG was lower in HWS. *Selenomonas_*1 was also higher in HWS. Rumen *Selenomonas_*1 is closely related to fiber digestion, and mainly produces PA [81]. Since this bacterium is also a low-abundance rumen bacterium, the PA content did not change significantly. However, there was a positive correlation between *Treponema_2* and OMD. Notably, *Treponema*_2 and *Selenomonas_*1 in the rumen is positively correlated with the vast majority of metabolites (e.g., ethanol, AA, and PA) and biomarker bacteria (e.g., *Lactiplantibacillus*) in WSS. Meanwhile, *Treponema*_2 in the rumen was positively correlated with the majority of silage biomarker microorganisms in WSS. This suggests that metabolites and biomarker bacteria in WSS may promote nutrient degradation by modulating key bacteria in the rumen, but without affecting the production of short-chain fatty acids in the rumen.

In this study, the dominant fungal phyla in the rumen of buffaloes were *Ascomycota*, *Neocallimastigomycota*, and *Basidiomycota* in both LWE and HWS. The same phyla are also found in the rumen of Simmental crossbreed bulls [82]. However, the relative abundance of the phylum *Ascomycota* was higher in HWS than in LWE (93.76% vs. 89.30%). *Ascomycetes* are a large group of fungi that are important drivers of nutrient cycling and are involved in lignin and keratin degradation [83,84]. The dominant fungal genus in this study was *Meyerozyma,* which is consistent with previous findings [82]. Kuo et al. [85] note that *Meyerozyma* sp. can secrete glycosidases, hydrolases, or specific cellulases. Moreover, some species of *Meyerozyma* yeast are potential probiotic strains [86,87]. The abundance of some other yeasts such as *Saccharomyces* was also higher in HWS than in LWE. *Saccharomyces* is also considered a probiotic, especially *S. cerevisiae*, which can effectively improve nutrient digestibility (e.g., DMD, OMD) [88]. However, the presence of yeast *Saccharomyces* sp. (e.g., *S. cerevisiae*) may reduce the activity of fibrolytic and amylolytic enzymes by modifying the abundance of the protozoan genera *Diplodinium* and *Dasytricha* in the rumen of sheep [89]. This may also be the reason why HWS has a lower DMD, OMD, and ADFD (Table 3). In addition, a low-abundance yeast, *Pseudozyma*, belonging to *Basidiomycota*, was higher in LWE. Studies have shown that some strains of the *Pseudozyma* can produce highly active xylanase [90,91]. Moreover, Mantel analysis also showed a positive correlation between *Pseudozyma* and ADFD. This may be a reason why LWE has a higher ADFD (Table 3). Meanwhile, the BA and the vast majority of biomarker microorganisms (e.g., *Clostridium_sensu_stricto*_11) in EGS also showed a positive correlation with *Pseudozyma.* This suggests that biomarker microorganisms and their metabolites in EGS may promote nutrient degradation by modulating key fungi in the rumen. However, most of silage biomarker microorganisms in EGS belong to *Clostridiaceae* or *Enterobacteriaceae*, which are potentially pathogenic bacteria that may threaten the health of animals.

## 5. Conclusions

In conclusion, compared with EGS, WSS had higher WSC, LA and ethanol contents, but lower pH, ammonia nitrogen, PA, and BA contents. Meanwhile, the potential beneficial microbial taxa (e.g., *Lactiplantibacillus* and *Hanseniaspora*) were more likely to proliferate in WSS, whereas undesirable microbes (e.g., *Enterobacter*) were more likely to grow in EGS. Moreover, the FCR was lower in LWE. However, no significant differences in rumen fermentation or dominant microbial communities were found between LWE and HWS. Notably, the HWS group showed lower apparent digestibility of DM, OM, and ADF, yet the low-abundance key microbes *Treponema*_2 and *Selenomonas*_1 in HWS were closely related to DM and ADF digestibility, as well as to these potential beneficial microbial taxa and organic acids in WSS. In contrast, in LWE, *Ruminiclostridium*_5 and *Pseudozyma* were associated with DMD and ADFD, respectively, while *Pseudozyma* was also linked to the pathogenic *Clostridium_sensu_stricto*_11 and its metabolite BA in EGS. In summary, although EGS resulted in higher nutrient digestibility in buffalo, it also contained potentially harmful bacteria; conversely, WSS had better fermentation quality and potential beneficial microbes but lower digestibility. Both advantages and limitations should be considered in practice.

## Figures and Tables

**Table 1:** Chemical composition of fresh elephant grass and whole-plant sugarcane.

Items ^1^	Elephant Grass	Whole-Plant Sugarcane
DM (g/kg FM)	269.94	294.95
CP (g/kg DM)	82.90	85.48
NDF (g/kg DM)	723.24	612.35
ADF (g/kg DM)	430.17	366.67
Hemicellulose (g/kg DM)	293.07	245.68
Ash (g/kg DM)	92.59	23.09
WSC (g/kg DM)	115.34	369.94
LAB (lg CFU/g FM)	2.34	1.94
EB (lg CFU/g FM)	2.58	3.18
Yeast (lg CFU/g FM)	2.87	2.39
Mold (lg CFU/g FM)	1.41	1.64

^1^ DM, dry matter; CP, crude protein; WSC, water-soluble carbohydrate; NDF, neutral detergent fiber; ADF, acid detergent fiber; FM, fresh matter; CFU, colony-forming units; LAB, lactic acid bacteria; EB, enterobacteria.

**Table 2:** Ingredients and chemical composition of experimental diets for water buffaloes based on 50% elephant grass silage (LWE) or 50% whole-plant sugarcane silage (HWS) (g/kg, on DM basis).

Items	Dietary Treatment	
	LWE ^1^	HWS ^2^
Elephant grass silage	500	
Sugarcane silage		500
Corn	100	100
Cassava meal	150	150
Corn bran	100	100
Soybean meal	80	80
Distiller’s grains	50	50
Salt	1.5	1.5
Soda	1.0	1.0
Premix ^3^	17.5	17.5
Chemical composition		
Gross energy (Mcal/kg)	4.20	4.13
Dry matter	585.78	578.90
Crude protein	118.62	101.35
Neutral detergent fiber	446.20	440.96
Acid detergent fiber	265.76	254.09
Ash	77.10	69.90
Organic matter	922.87	930.08

^1^ LWE = fed basic diet supplemented with 50% elephant grass silage. ^2^ HWS = fed basic diet supplemented with 50% whole-plant sugarcane silage. ^3^ The composition of the premix is as follows: VA 500 KIU/kg; VD3150 KIU/kg; VE 3000 IU/kg; Fe 4.0 g/kg; Cu 1.3 g/kg; Mn 3.0 g/kg; Zn 6.0 g/kg; I 80 mg/kg; Se 50 mg/kg; and Co 80 mg/kg.

**Table 3:** Chemical composition and fermentation quality of elephant grass silage (EGS) and whole-plant sugarcane silage (WSS).

Items	EGS ^1^	WSS ^2^	SEM ^3^	*p*-Value
Chemical composition ^4^, g/kg DM				
DM	258.76	244.99	0.343	0.02
CP	63.33	64.35	0.299	0.02
WSC	31.88	88.44	16.365	<0.01
NDF	698.88	688.40	27.463	0.89
ADF	439.20	415.86	22.864	0.71
Hemicellulose	259.69	272.54	17.365	0.47
Microbial counts ^5^, log CFU/g FM				
LAB	2.34	1.94	0.133	0.14
EB	2.58	3.18	0.172	<0.01
Yeasts	2.87	2.39	0.145	0.02
Molds	1.41	1.64	0.077	0.11
Fermentation index ^6^, g/kg DM				
pH	5.18	3.39	0.342	0.04
LA	23.07	29.73	1.777	0.01
NH3-N	0.15	0.06	0.021	0.17
Ethanol	8.20	22.31	3.688	0.03
AA	8.60	7.65	1.257	0.75
PA	2.03	1.53	0.124	0.01
BA	2.97	1.54	0.382	0.04

^1^ EGS, elephant grass silage. ^2^ WSS, whole-plant sugarcane silage. ^3^ SEM, standard error of the mean. ^4^ DM, dry matter; CP, crude protein; WSC, water-soluble carbohydrate, NDF, neutral detergent fiber; ADF, acid detergent fiber; OM, organic matter. ^5^ FM, fresh matter; CFU, colony-forming units; LAB, lactic acid bacteria; EB, enterobacteria. ^6^ LA, lactic acid; AA, acetic acid; PA, propionic acid; BA, butyric acid. *p* < 0.05 is considered statistically significant.

**Table 4:** Effects of fed elephant grass silage (LWE) and whole-plant sugarcane silage (HWS) on growth performance, apparent nutrient digestibility, and rumen fermentation of buffaloes.

Items	LWE ^1^	HWS ^2^	SEM ^3^	*p*-Value
Growth performance ^4^				
Initial BW (kg)	546.79	528.57	8.690	0.35
Final BW (kg)	559.57	535.71	7.173	0.21
ADG (kg/d)	0.32	0.18	0.039	0.35
DMI (kg/d)	12.07	11.20	0.205	0.24
FCR	37.73	61.53	3.656	<0.001
Apparent nutrient digestibility ^5^				
DMD (%)	55.16	47.39	1.019	<0.001
OMD (%)	57.25	49.89	0.913	<0.001
CPD (%)	43.35	39.25	1.527	0.19
NDFD (%)	42.62	40.26	0.913	0.20
ADFD (%)	43.77	30.58	1.546	<0.001
GED (%)	48.83	47.68	0.575	0.33
Rumen fermentation ^6^				
pH	6.73	6.72	0.016	0.53
NH3-N (mg/dL)	3.85	3.72	0.253	0.82
AA (mmol/L)	83.36	87.26	3.652	0.61
PA (mmol/L)	20.60	20.10	1.210	0.14
BA (mmol/L)	9.57	11.29	0.833	0.18

^1^ LWE, fed basic diet supplemented with 50% elephant grass silage. ^2^ HWS, fed basic diet supplemented with 50% whole-plant sugarcane silage. ^3^ SEM, standard error of the mean. ^4^ ADG, average daily gain; DMI, dry matter intake; FCR, feed conversion rate. ^5^ DMD, dry matter digestibility; CPD, crude protein digestibility; NDFD, neutral detergent fiber digestibility; ADFD, acid detergent fiber digestibility, OMD, organic matter digestibility; GED, gross energy digestibility. ^6^ AA, acetic acid; PA, propionic acid; BA, butyric acid.

## Data Availability

Bacterial and fungal raw sequencing data of the silage samples have been deposited in the NCBI (https://www.ncbi.nlm.nih.gov/, accessed on 30 July 2024) BioProject database with accession numbers PRJNA 1289997 and PRJNA1289998, respectively, and those of the ruminal fluid samples also have been deposited with accession numbers PRJNA1141254 and PRJNA1141256, respectively.

## Supplementary Materials

The following supporting information can be downloaded at https://www.mdpi.com/article/10.3390/ani16081233/s1: Table S1: Library coverage of bacteria and fungi in elephant grass silage (EGS) and whole-plant sugarcane silage (WSS); Table S2: Library coverage of bacteria and fungi in rumen.

## Abbreviations

The following abbreviations are used in this manuscript:

DM	Dry matter
FM	Fresh matter
CP	Crude protein
WSC	Water-soluble carbohydrates
OM	Organic matter
NDF	Neutral detergent fiber
ADF	Acid detergent fiber
CFU	Colony-forming units
LAB	Lactic acid bacteria
EB	enterobacteria
LA	Lactic acid
AA	Acetic acid
PA	Propionic acid
BA	Butyric acid
GE	Gross energy
BW	Body weight
ADG	Average daily gain
DMI	Dry matter intake
FCR	Feed conversion rate
DMD	Dry matter digestibility
CPD	Crude protein digestibility
NDFD	Neutral detergent fiber digestibility
ADFD	Acid detergent fiber digestibility
OMD	Organic matter digestibility
GED	Gross energy digestibility.
DNA	Deoxyribonucleic acid
RNA	Ribonucleic acid
OTU	Operational taxonomic units
LEfSe	Linear discriminant analysis effect size
SEM	Standard error of the mean
