# Supplementary material for: The Effects of C4 Forage Silage with Different Water-Soluble Carbohydrate Contents on the Growth Performance, Apparent Digestibility, Rumen Fermentation, and Rumen Microbial Community of Buffaloes
Source: Animals (Basel). 2026 Apr 17;16(8):1233. doi: 10.3390/ani16081233 (PMC13114166; doi:10.3390/ani16081233)
Supplement: Supplementary file 1 [file animals-16-01233-s001.zip › animals-4197933-supplementary.pdf]

Table S1. Library coverage of bacteria and fungi in elephant grass silage (EGS) and whole-plant sugarcane silage (WSS).

Sample_Name	Bacteria	Fungi
WSS1	0.997	0.998
WSS2	0.997	0.999
WSS3	0.996	0.999
WSS4	0.998	0.999
EGS1	0.997	0.998
EGS2	0.996	0.998
EGS3	0.998	0.998
EGS4	0.997	0.999

Table S2. Library coverage of bacteria and fungi in rumen.

Sample name[1]	Bacteria	Fungi
LWE1	0.996	0.998
LWE2	0.997	0.999
LWE3	0.997	0.999
LWE4	0.996	0.999
LWE5	0.996	0.998
LWE6	0.997	0.998
LWE7	0.998	0.998
HWS1	0.997	0.999
HWS2	0.995	0.998
HWS3	0.997	0.999
HWS4	0.996	0.998
HWS5	0.997	0.999
HWS6	0.998	0.998
HWS7	0.997	0.999

[1]LWE, Fed basic diet supplemented with 50% elephant grass silage. 2HWS, Fed basic diet supplemented with 50% whole-plant sugarcane silage.
